# Phantom-Based Approach for Comparing Conventional and Optically Pumped Magnetometer Magnetoencephalography Systems

**DOI:** 10.3390/s25072063

**Published:** 2025-03-26

**Authors:** Daisuke Oyama, Hadi Zaatiti

**Affiliations:** 1 Applied Electronics Laboratory, Kanazawa Institute of Technology, Kanazawa 920-1331, Japan; 2 Bio-Medical Imaging Core, Core Technology Platforms, New York University Abu Dhabi, Abu Dhabi P.O. Box 129188, United Arab Emirates; hadi.zaatiti@nyu.edu

**Keywords:** magnetoencephalography, superconducting quantum interference device, optically pumped magnetometers, dry phantom

## Abstract

Magnetoencephalography (MEG) is a vital tool for understanding neural dynamics, offering a noninvasive technique for measuring subtle magnetic field variations around the scalp generated by synchronized neuronal activity. Two prominent sensor technologies exist: the well-established superconducting quantum interference device (SQUID) and the more recent optically pumped magnetometer (OPM). Although many studies have compared these technologies using human-subject data in neuroscience and clinical studies, a direct hardware-level comparison using dry phantoms remains unexplored. This study presents a framework for comparing SQUID- with OPM-MEG systems in a controlled environment using a dry phantom that emulates neuronal activity, allowing strict control over physiological artifacts. Data were obtained from SQUID and OPM systems within the same shielded room, ensuring consistent environmental noise control and shielding conditions. Positioning the OPM sensors closer to the signal source resulted in a signal amplitude approximately 3–4 times larger than that detected by the SQUID-MEG system. However, the source localization error of the OPM-MEG system was approximately three times larger than that obtained by the SQUID-MEG system. The cause of the large source localization error was discussed in terms of sensor-to-source distance, sensor count, signal–noise ratio, and the spatial coverage provided by the sensor array of the source signal.

## 1. Introduction

Magnetoencephalography (MEG) [1] measures the magnetic fields generated by neural activity, offering high temporal and spatial resolution for studying brain dynamics, compared to functional magnetic resonance imaging and electroencephalography, respectively. Superconducting quantum interference device (SQUID)-MEG has been widely used for decades because of its high sensitivity. SQUID sensors require cryogenic cooling to detect subtle magnetic signals like 100 femtotesla. These sensors are kept in a fixed position within a dewar, which is filled with cryogens, typically at approximately 4 K. The need for large and rigid dewars to maintain cryogenic conditions often results in a sensor-to-scalp distance of 2–3 cm, which weakens the measured signal. The increased distance reduces the signal–to–noise ratio (SNR), potentially degrading the spatial resolution of source localization.

Over the past decade, improvements in laser stability, vapor-cell fabrication, and magnetic shielding have improved the practicality of optically pumped magnetometers (OPMs) for on-scalp MEG. By 2010, prototype OPM-MEG systems emerged, including wearable arrays that allow free-head movement, positioning this technology as an alternative or complement to SQUID-MEG [2,3]. OPM technology offers a more cost-effective and wearable solution, unlike the SQUID system, which relies on costly and volatile cryogen supply chains. As OPM sensors operate without cryogens, they can be positioned closer to the scalp, reducing the sensor-to-scalp distance and accounting for the head movements of individuals. Additionally, OPM systems feature a flexible sensor layout, allowing optimized sensor placement for specific head regions of interest. The bulky dewar helmet of SQUID systems limits participant movement, posing challenges for studies involving children, clinical populations with movement disorders, and motion-dependent experiments. Rapid advancements in OPM technology have expanded biomagnetic applications, including MEG applications [4].

This study compares the two technologies crucial for advancing MEG applications in both research and clinical practice. A hardware-level comparison presents significant challenges, as it requires acquiring data under identical conditions while accounting for the unique capabilities of each system. Key factors include environmental noise and physiological brain activity, which may introduce artifacts unrelated to the task. Since these systems share the same purpose but use different underlying technologies, an effective comparison requires identifying a transformation that captures the strength of each technology and allows for comparison within a common framework.

### 1.1. Comparison Studies on SQUID-MEG and OPM-MEG Systems

Several studies have explored the comparison between SQUID-MEG and OPM-MEG systems. For instance, Hill et al. built whole-head OPM systems that provide whole-head coverage [5]. The authors used a visuo-motor paradigm, acquiring data from their constructed 49-sensor helmet using both flexible and rigid helmets. The same paradigm was also recorded using a SQUID-MEG system with 275 gradiometers. Each participant underwent 18 scans, and the comparison was performed using the beamformer-based source reconstruction approach. Several metrics were used to evaluate sensor-space data, source-space reconstructed images, and source-space time series of neuronal activity. Marhl et al. recently compared the SQUID-MEG and OPM-MEG data of auditory-evoked fields [6,7]. Their study proposed a method for transforming data between MEG systems by computing the forward model of one system and applying it to the inverse problem of a second system. Unlike Hill et al. [5], who focused on source-space analysis, Marhl et al. [6] used the sensor space. Additionally, Nugent et al. performed a comparison based on simulation [8].

### 1.2. Phantom-Based Studies

To quantitatively evaluate the MEG system performance, we developed a dry phantom that simulates neuronal activity in the human brain [9]. This phantom comprises 50 triangular coils that generate small currents analogous to human brain dipole currents, known as equivalent current dipoles (ECDs). Analyzing the measured data from these coils allows for evaluating source localization accuracy and validating technological advancements in MEG systems [10,11]. Furthermore, multiple phantom-based MEG studies have demonstrated advanced data processing and localization methods. Multi-dipole phantoms, global-to-local optimization algorithms, and empirical mode decomposition have enhanced inverse solutions under challenging conditions [12,13,14]. Several phantom experiments have also addressed specialized scenarios, including deep brain stimulation hardware interference and deeper or more complex source configurations, demonstrating the adaptability of MEG with proper artifact suppression and modeling [15,16]. Finally, integrating anatomical imaging into phantom experiments significantly reduces registration errors and enhances the overall localization accuracy, confirming the importance of structural–functional alignment in MEG performance evaluation [17,18]. Recently, OPMs have demonstrated substantial potential alongside traditional SQUIDs. These innovations were validated in controlled phantom experiments, as highlighted by Ito et al. [19]. Boto et al. [20] explored a triaxial OPM capable of measuring the full three-dimensional vector of neuromagnetic fields. This system was validated through wet phantom dipole simulations and in vivo measurements, exhibiting high precision with a minimal source-localization error of 5.17 mm. More recently, Bardouille et al. evaluated a cylindrical-shielded OPM-MEG system using a dry phantom, demonstrating that this system achieves approximately 3 mm localization error without significant bias, comparable to cryogenic SQUID and other OPM-MEG systems [21].

### 1.3. Motivation and Study Outline

This study is driven by the effectiveness of phantom-based approaches in measuring MEG system performances, alongside the absence of a hardware-focused comparison of SQUID and OPM systems. We propose a comparison framework in a highly controlled setup between SQUID-MEG and OPM-MEG systems to address this gap, employing a dry phantom as described in [9], owing to its well-characterized geometry and metallic conductivity which ensures greater measurement certainty than a wet phantom. A wet phantom provides a more human-like brain model, which introduces more uncertainty, as measuring the electric current path is more challenging. As this study focuses on the hardware-centric comparison of SQUID-OPM technologies, a dry phantom provides more reliable and controlled environments. As mentioned in [22], insulating structures within a wet phantom can distort volume currents near the dipole, potentially compromising their effectiveness for absolute calibration. This is particularly problematic when the phantom is the ground truth for assessing source localization accuracy. Our primary objective is to compare SQUID-MEG and OPM-MEG systems while minimizing confounding factors, including non-task-related brain activity like eye movements and cardiac artifacts, shielding properties of the magnetically shielded room (MSR), and noise from nearby equipment or infrastructure, especially when the systems are not co-located. We eliminated subject variability and physiological artifacts by employing a carefully designed coil-based phantom to address these issues, ensuring consistency across the SQUID-MEG and OPM-MEG measurements. Additionally, we standardized environmental conditions by acquiring data for both systems within the same MSR on the same day, thereby controlling external noise. Moreover, this framework enables quantitative validation by establishing a ground-truth benchmark that can be applied to various MEG setups, including those from different manufacturers worldwide.

This paper is organized as follows: Section 2 details the methodology for comparing SQUID-MEG and OPM-MEG systems using phantom-based measurements and analyzing the acquired data. Section 3 presents the results, focusing on source localization accuracy by comparing calibrated and source-reconstructed ECDs. Finally, Section 4 and Section 5 discuss the results, concluding and drawing future perspectives.

## 2. Methods

### 2.1. SQUID-MEG and OPM-MEG Systems

**Figure 1:**
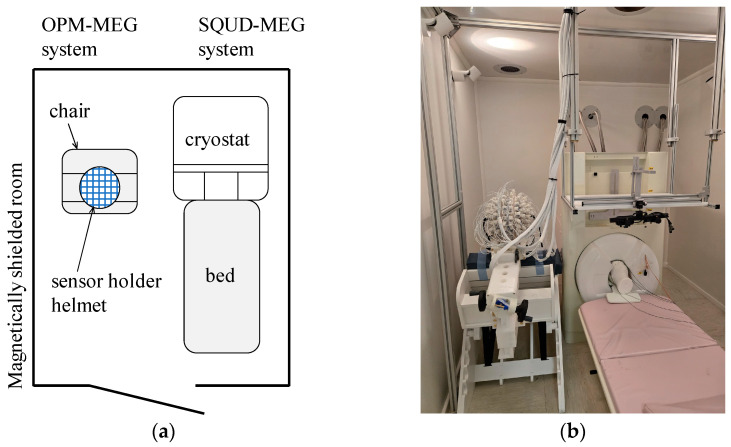
Configuration of the SQUID- and OPM-MEG systems in a single MSR: (**a**) schematic of the measurement setup; (**b**) photograph of the setup at NYUAD.

Experiments were performed using both the SQUID-MEG and OPM-MEG systems. Both the MEG systems were installed next to each other in an MSR at New York University, Abu Dhabi (NYUAD). Figure 1 shows the schematic and photograph of the experimental setup. The SQUID-MEG system was installed on the right side of the MSR and could not be moved. Therefore, the OPM-MEG system was placed on the left side of the room.

The SQUID-MEG system provided and installed by Eagle Technology, Inc. (Kanazawa, Japan) comprised an array of 208 first-order axial gradiometer SQUID sensors, following the same fundamental design as described in [23]. The SQUID sensors were arranged to cover the dewar helmet (Arisawa Manufacturing Co., Ltd., Joetsu, Japan). In addition to the gradiometers used to detect the brain signal, 16-channel SQUID magnetometers were also installed in the same dewar. These SQUID magnetometers were positioned apart from the helmet to measure only magnetic noise, thereby facilitating the application of noise suppression algorithms such as the continuously adjusted least squares method [24] or time-shifted principal component analysis [25].

The OPM-MEG system used in this study was the HEDscan system (Fieldline Medical Inc., Boulder, CO, USA) [26], which allows flexible sensor arrangement through a helmet-mounted holder. In this study, 96-channel OPM sensors were installed, with the specific arrangement detailed in Section 2.3. All the SQUID and OPM sensors used in this study detected the radial component of the magnetic field distribution.

**Figure 2:**
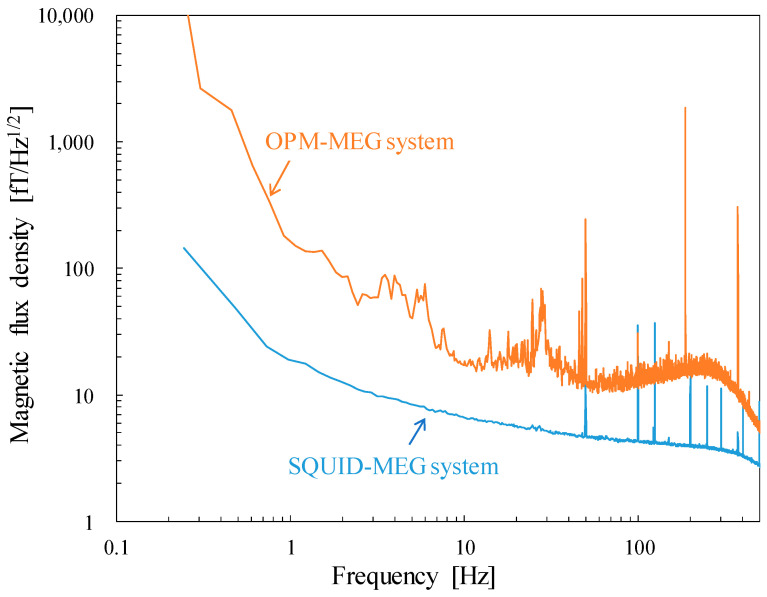
Noise spectra of the SQUID- and OPM-MEG systems.

Figure 2 shows the noise spectra measured without subjects in the MEG system. These datasets were simultaneously recorded for 30 s with sampling frequencies of 2 kHz and 5 kHz using the SQUID- and OPM-MEG systems, respectively. During the recordings, the following online filters were applied: a 0.03 Hz high-pass and a 500 Hz low-pass filter in the SQUID-MEG system, and a 500 Hz low-pass filter in the OPM-MEG system. During initialization, some sensors in both systems failed or exhibited excessive noise; therefore, we used 202 SQUID sensors and 90 OPM channels. The spectra were obtained as the mean of 202- and 90-channels except for the operation failure or noisy channels. The measured noise floors were 4 fT/Hz^1/2^ at 180 Hz for the SQUID sensors and 12 fT/Hz^1/2^ at 70 Hz for the OPM sensors. There are several significant differences between the two spectra besides the difference in the noise floors which are typically determined by the intrinsic noise level of each sensor. The power line noise of the SQUID-MEG system at 50 Hz is approximately 1/20 of that of the OPM-MEG system, as the SQUID-MEG employs the gradiometers. The noise originated from a vibration that appeared below 30 Hz in the OPM-MEG system. The SQUID-MEG system is more robust to the vibration because the SQUID sensors are fixed to the dewar and equipped with gradiometric pickup coils. The noise floor of the OPM-MEG system was slightly elevated above 100 Hz. Based on its characteristic shape, this increase is attributed to the frequency response of the OPM sensors, which have a cut-off frequency of approximately 300 Hz. Additionally, sharp and prominent peaks above 100 Hz are observed in both MEG systems—for instance, at 125 Hz in the SQUID-MEG and at 187 Hz in the OPM-MEG systems. These noises are not commonly observed in the two MEG systems; therefore, these noises seemingly penetrated electrically through the power or signal lines of each device, originating from an unidentified device.

### 2.2. Phantom

**Figure 3:**
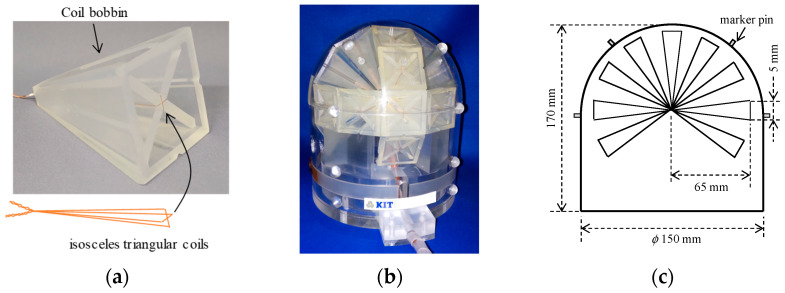
Photograph and configuration of the dry-type MEG phantom: (**a**) closeup view of the triangular coil; (**b**) photograph of the dry-type MEG phantom; (**c**) schematic and dimensions of the phantom.

We employed a dry-type MEG phantom to compare the performance of the two MEG systems [9], which simulate neuronal activity in the human brain through ECDs. Figure 3 shows the configuration of the phantom. This phantom comprises 50 isosceles triangular coils that generate a magnetic field pattern similar to that of an ECD. Each bobbin contains two perpendicularly wound coils, as shown in Figure 3a. Twenty-five bobbins were assembled to be spherical, as shown in Figure 3b. The isosceles triangular coils had a height of 65 mm and a base of 5 mm, ensuring that each ECD was placed on the surface of an imaginary conductive sphere with a radius of approximately 65 mm. Figure 3c shows the dimensions of the phantom. The coil bobbins were fixed inside a domed cover, with the phantom measuring 170 and 150 mm in height and diameter, respectively. Five small cylindrical pins on the surface of the phantom allowed for the attachment of marker coils for the co-registration of the phantom and MEG coordinate systems.

### 2.3. Measurements

**Figure 4:**
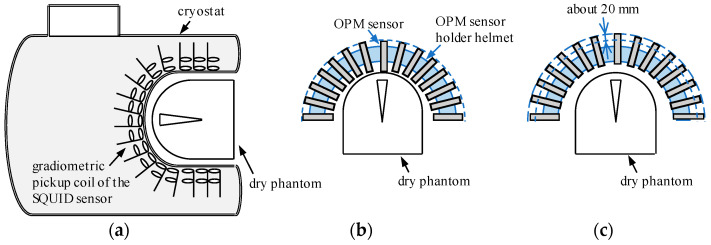
Experimental setup with the dry phantom: (**a**) SQUID-MEG system; (**b**) OPM-MEG system (closest position); (**c**) OPM-MEG system (lift-off position).

The dry phantom was positioned at the center of each sensor array during the experiments, as illustrated in Figure 4. For the SQUID-MEG system, the phantom was placed at the center of the cryostat helmet (Figure 4a). The sensor-holder helmet was held by a chair in the OPM-MEG setup. First, we inserted the OPM sensors fully into the sensor holder and then adjusted the height of the sensor holder to be as close as possible to the phantom, as shown in Figure 4b. Next, the OPM sensors were shifted outward by approximately 20 mm while keeping the helmet and phantom stationary, as shown in Figure 4c. As described in Section 1, the advantage of OPM sensors is their ability to be positioned closer to the signal source than SQUID sensors. SQUID-MEG systems have a lift-off distance of 20 mm owing to the insulation layer [23]. In this study, we compared the signal amplitude and source localization accuracy of an OPM-MEG system at the closest and lift-off positions. Therefore, we conducted measurements using three sensor arrays, as shown in Figure 4a–c. All the measurements were completed in a day to ensure consistent environmental magnetic noise.

The spatial relationship between the sensor arrays and the phantom was measured using marker coils, also called head position indicator coils—a technique introduced by Erné [27]. Five marker coils were attached to the cylindrical pins on the surface of the phantom. Each coil was activated separately with an alternating current, generating a magnetic field measured by the MEG systems. The positions of the marker coils were estimated by solving an inverse problem, assuming the coils were magnetic dipoles. Rigid body transformation matrices between the phantom and MEG sensor array coordinate systems were derived based on the localized positions of the measured marker coils. The frequency of the applied current to the marker coils was 80 Hz and 43 Hz for the SQUID- and OPM-MEG systems, respectively. Details of the marker coils and current application patterns are described in Appendix A.

**Figure 5:**
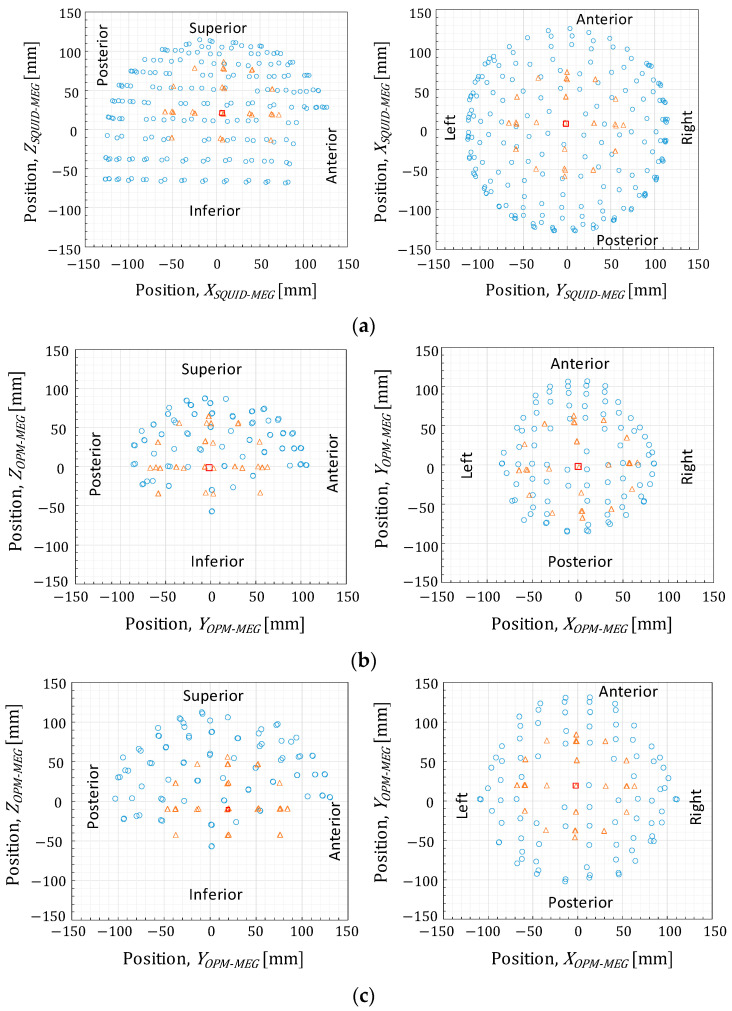
Positions of the sensors (circles), effectual ECDs (triangles), and center of the conductive sphere model (squares): (**a**) SQUID-MEG system; (**b**) OPM-MEG system (closest position); (**c**) OPM-MEG system (lift-off position).

Figure 5 shows the positions of the sensors and ECDs in the MEG coordinate system. The ECD positions do not correspond to the triangular coil positions but to the effectual ECD positions calibrated using X-ray computed tomography and numerical calculations, as described in [9].

**Figure 6:**
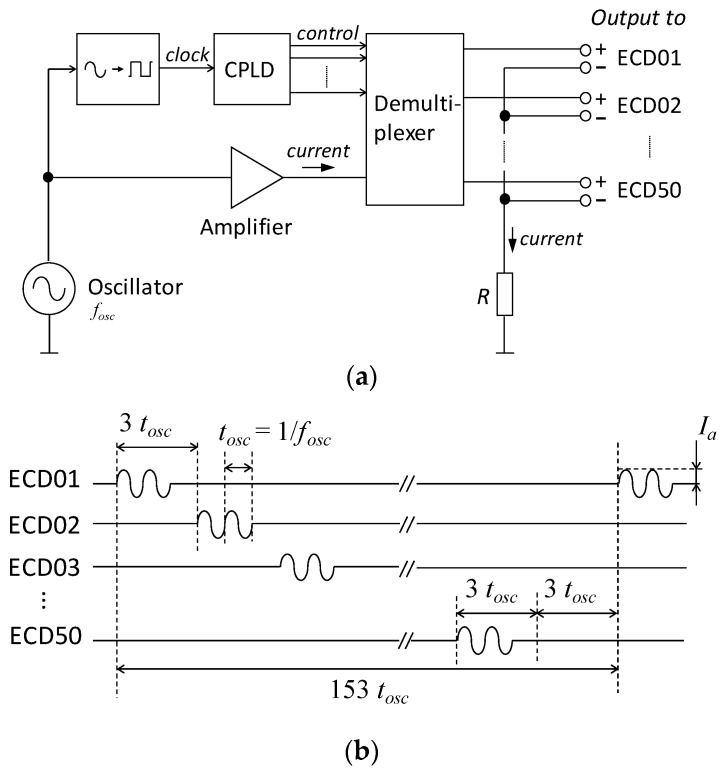
A current-driver circuit for the dry phantom: (**a**) schematic diagram; (**b**) waveforms of the current sequentially applied to the phantom.

After measuring the marker coil, the phantom was connected to a current-driver circuit that sequentially applied a sinusoidal current to each coil. Figure 6 presents a simplified block diagram of the current-driver circuit along with its output current waveform. The circuit has fifty output channels, which are connected to the triangular coils of the dry phantom. All the positive terminals of the output channels are connected to an amplifier through a demultiplexer, which is switched by control signals generated by a complex programmable logic device (CPLD). Using the output signal of an oscillator as a clock for the CPLD enables the output channel to switch in synchronization with the sinusoidal waveform of the output current, as shown in Figure 6b. *t_osc_* in Figure 6b represents the reciprocal of the frequency of the oscillator *f_osc_*, which is set to 11 Hz in accordance with the previous study [9].

First, the applied current *I_a_* was set to 10 µA, resulting in an ECD moment intensity of approximately 50 nA·m, emulating neuronal activity in the human brain. Next, *I_a_* was set to 100 µA to assess the source localization accuracy with an improved SNR. Data were recorded for 840 s to capture more than 50 repetitions of the current supply sequence, with 2 and 5 kHz sample frequencies in the SQUID- and OPM-MEG systems, respectively.

### 2.4. Analysis

The recorded data were first down-sampled to 1 kHz to satisfy the analysis conditions. A 0.5 Hz high-pass filter was then applied offline, followed by 50 times averaging to reduce magnetic noise. The magnetic field distributions at the first peaks of each ECD signal were captured, and the signal sources were estimated using the Sarvas formula [28], as in previous studies [9,29]. Corresponding to the sensor structures, we employed gradiometer and magnetometer models to solve the inverse problem with the data from the SQUID- and OPM-MEG systems, respectively. The discrepancy between the estimated and effectual ECDs was considered the source estimation error. In this study, we focused on the distance between the estimated and effectual ECDs to simplify the discussion.

## 3. Results

**Figure 7:**
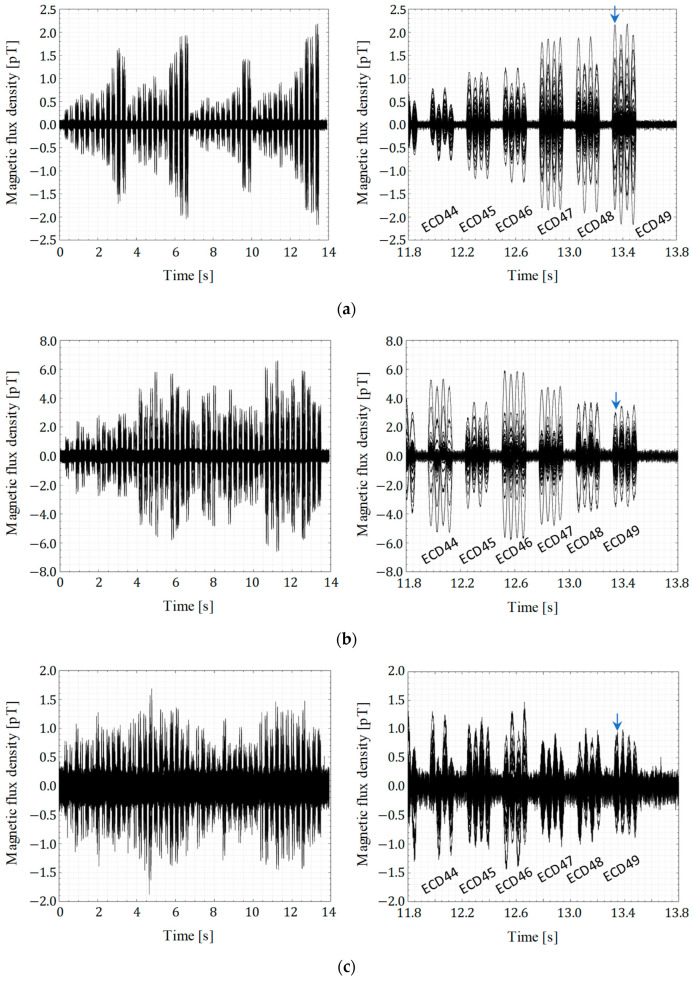
Butterfly plot of recorded signals from SQUID- and OPM-MEG sensors during the activation of the triangular phantom coils (ECD 1–49): (**a**) SQUID-MEG system; (**b**) OPM-MEG system (closest position); (**c**) OPM-MEG system (lift-off position). The arrows indicate the analysis timing for ECD 49 as represented in Figure 9.

Figure 7 shows the recorded waveforms with the current amplitude *I_a_* of 10 µA after applying a high-pass filter and averaging. Figure 7a shows data recorded by the SQUID-MEG system. Figure 7b,c display data from the OPM-MEG system with the closest and lift-off positions, respectively. Each graph contains overlapping waveforms corresponding to individual sensors—202 and 90 waveforms for the SQUID- and OPM-MEG systems, respectively. The right column provides a magnified view of a specific time window from the corresponding graphs in the left column, focusing on the signals recorded during the activation of ECD44 to ECD49. As shown in these graphs, the signal from coil 50 disappeared because of accidental damage to its wiring. The sinusoidal waveforms generated by the remaining coils were obtained. The maximum amplitude of the observed signals was approximately 2.2 pT, 6.6 pT, and 1.7 pT in Figure 7a, Figure 7b, and Figure 7c, respectively.

**Figure 8:**
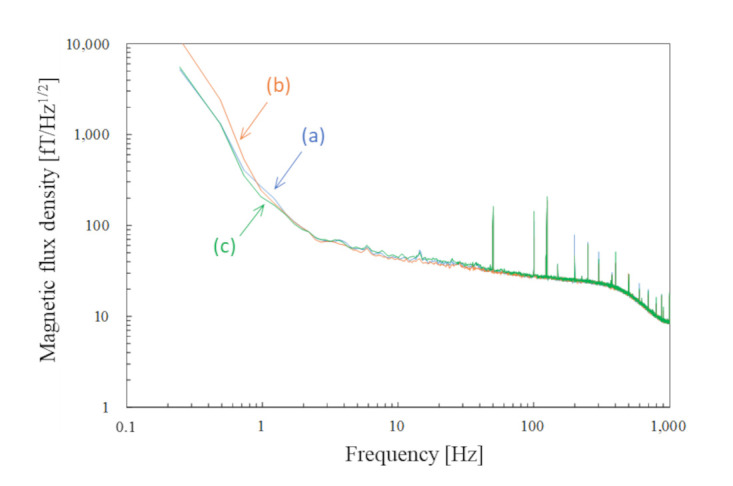
Noise spectra recorded by the SQUID magnetometers simultaneously with the phantom measurements using (**a**) SQUID-MEG system; (**b**) OPM-MEG system (closest position); (**c**) OPM-MEG system (lift-off position).

As described in Section 2.3, all the measurements were completed in a day. To confirm the consistent environmental magnetic noise, we compared the magnetic noise recorded by the SQUID magnetometers simultaneously with the phantom signal shown in Figure 7. A 3-axis SQUID vector magnetometer [30] located 250 mm away from the top of the SQUID gradiometers was selected from the 16-channel SQUID magnetometers installed in the SQUID-MEG system. The noise spectra presented in Figure 8 were obtained as the mean of the 3-channel SQUID magnetometers during the measurement. Figure 8a–c correspond to those in Figure 7. The noise trend of Figure 8b, recorded during the OPM-MEG measurement at the closest sensor position, is slightly higher than that of the others below 1 Hz. This increase is presumed to be caused by the movement of magnetic objects, such as cars or elevators. However, this difference is considered negligible because we applied the high-pass filter before analyzing the data of the phantom measurements. No noticeable differences above 1 Hz were observed across the measurements, despite an approximately 5 h interval between the first and last recordings.

**Figure 9:**
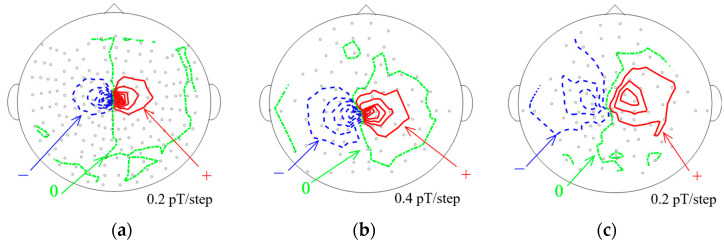
Magnetic field distribution from the 49th ECD recorded by each sensor configuration: (**a**) SQUID-MEG system; (**b**) OPM-MEG system (closest position); (**c**) OPM-MEG system (lift-off position). Small circles indicate the relative position of the sensors.

Figure 9 shows the magnetic field distribution generated by ECD49, located at the top of the phantom, with the current amplitude *I_a_* of 10 µA. The small circles in this figure indicate the relative positions of the sensors. The positive (+) and negative (−) signs are defined as outward and inward orientations, respectively. The data analysis timing corresponds to the first peak of the sinusoidal wave. Figure 9a–c correspond to those in Figure 7. A dipole pattern appears in all the datasets, but the magnetic field distribution differs noticeably. The area where the magnetic signal is observed expands in the order of Figure 9a–c. Furthermore, the distance between the sensors detecting the maximum and minimum signals varies.

**Figure 10:**
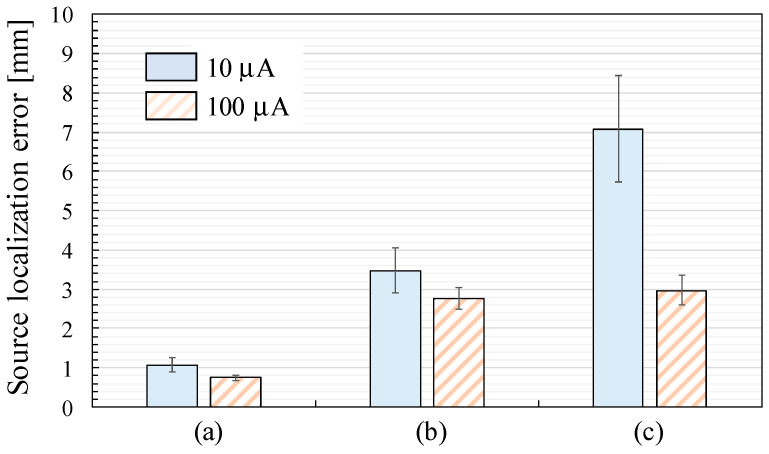
Source localization error defined as the displacement between the effectual and estimated ECD positions: (**a**) SQUID-MEG system; (**b**) OPM-MEG system (closest position); (**c**) OPM-MEG system (lift-off position).

Figure 10 shows the source localization error defined as the displacements between the effectual and estimated ECD positions with the current amplitude *I_a_* of 10 µA and 100 µA. Since coil 50 was disconnected during the recordings, the height of the squares and the length of the error bars indicate the mean and expanded uncertainty, respectively, with a coverage factor of two across 49 ECDs. Comparing the results at the current amplitude, the source localization error with *I_a_* = 100 µA is smaller than that with *I_a_* = 10 µA across all the sensor arrangements. The source localization error with *I_a_* = 10 µA increases in the order of Figure 10a–c.

## 4. Discussion

As described in Section 1, an advantage of OPM sensors is their ability to detect greater signal amplitude when positioned closer to the signal source. Comparing the two OPM sensor arrangements, the closest and lift-off positions, the observed signal amplitude in Figure 7b was approximately four times greater than that in Figure 7c. According to the Sarvas formula, the observable magnetic signal intensity is proportional to *d*^−2^, where *d* is the distance between the signal source and the observing point. The mean distances between each OPM sensor to the center of the conductive sphere model, indicated as circles and squares in Figure 5, were 90.1 and 114.3 mm in Figure 5b and Figure 5c, respectively. Assuming that the ECDs in the phantom were distributed on the surface of the sphere with a radius of 65 mm, the mean distances between the ECDs and OPM sensors were derived as 25.1 and 49.3 mm, respectively. The sensor-to-source distance and signal intensity relationship aligned well with the Sarvas formula. These findings, therefore, demonstrate the advantage of using OPM sensors.

Compared to the SQUID-MEG system, the OPM-MEG system recorded signal amplitudes up to three times higher, as shown in Figure 7. The mean distance between the ECDs and SQUID sensors was 54.6 mm, calculated by the method described above. The observed signal amplitude and sensor-to-source distance between the two MEG systems do not simply follow the *d*^−2^ proportionality because of structural differences; SQUID sensors use first-order axial gradiometers. Therefore, the waveform and magnetic field distributions differ between Figure 7a,b and Figure 9a,b. In the SQUID-MEG system, strong signals were detected near the signal source (Figure 8) because the gradiometer suppressed distant signals. Besides the increased signal amplitude from closer OPM sensor placement, it was found that the ratio of sensors detecting strong signals increased when using non-gradiometric detection sensors.

However, the estimated ECD displacement measured by the OPM-MEG system with the closest sensor position (3.48 ± 0.58 mm) in Figure 10b was larger than that measured by the SQUID-MEG system (1.07 ± 0.17 mm) in Figure 10a, despite the higher detected signal amplitude when the sensors were closer to the sources.

Several factors beyond signal intensity can limit source localization accuracy in MEG measurements. This study examined three major factors: SNR, the number of sensors used for localization, and the spatial coverage provided by the sensor array of the source signal.

First, the SNR is crucial to source estimation error. To investigate this relationship, we conducted recordings using a phantom signal with a ten-fold increase in applied current (100 µA) to increase the SNR in addition to the recording at 10 µA generating an ECD moment intensity emulating neuronal activity in the human brain. As shown in Figure 10, the resulting displacements were 0.74 ± 0.07 and 2.77 ± 0.29 mm with the SQUID- and OPM-MEG systems with the closest sensor positions, respectively. Although these displacements were smaller than those recorded at 10 µA, the OPM-MEG system still exhibited larger localization errors than the SQUID-MEG system. Therefore, insufficient SNR in the OPM-MEG system was not the reason for the larger source estimation error.

**Figure 11:**
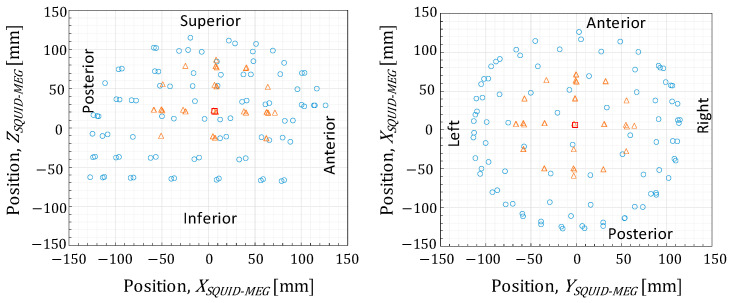
Selected 90-channel SQUID sensors for re-estimation. Circles, triangles, and squres indicate the positions of the sensors, effectual ECDs, and center of the conductive sphere model, respectively.

**Figure 12:**
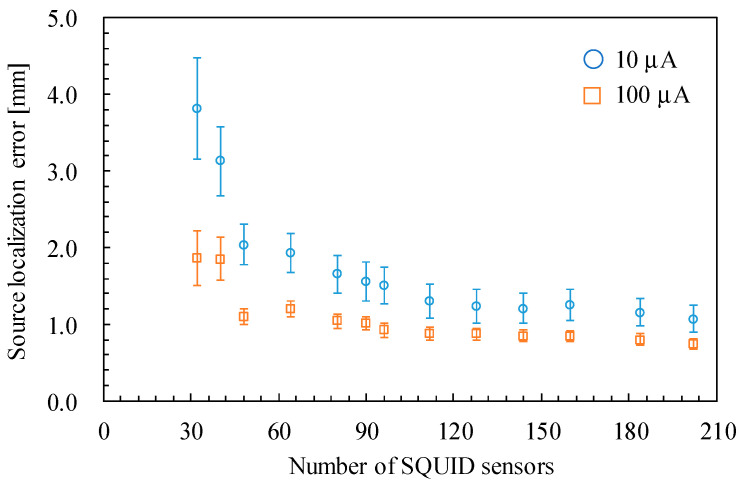
Dependence of the source localization error of the SQUID-MEG system on the number of sensors.

Second, the source localization accuracy depends on the number of sensors used for the estimation. As described in Section 2.3, the experiments used 202 and 90 sensors in the SQUID- and OPM-MEG systems, respectively. Although increasing the number of OPM sensors is expected to improve the source estimation accuracy, the maximum number of available OPM sensors in our laboratory was 90. Therefore, we re-estimated the ECDs using a different number of sensors in the SQUID-MEG system to examine the dependence of the source estimation accuracy on the number of sensors. We selected 32, 40, 48, 64, 80, 90, 96, 112, 128, 144, 160, and 184 sensors from the 202 sensors as uniformly as possible. For instance, Figure 11 presents the selected 90-channel sensor array. Figure 12 shows the source localization error with different numbers of sensors. The results for the 202-channel sensor array are identical to the data presented in Figure 10a. The source localization error increases as the number of sensors decreases, particularly when the number of sensors is fewer than 112. Focusing on the comparison between the SQUID-MEG and OPM-MEG systems, the displacements of the ECD localization with 90-channel SQUID sensors were 1.56 ± 0.25 and 1.01 ± 0.09 mm for the applied currents of 10 µA and 100 µA, respectively. Although these displacements were larger than those obtained using the 202-channel SQUID sensors, the OPM-MEG system exhibited larger displacements than those of the SQUID-MEG system. Therefore, insufficient OPM sensors was not the major cause of the larger source estimation error, even with a good SNR. Additionally, the displacement of the ECD localization with 40-channel SQUID sensors was 3.13 ± 0.45 mm at 10 µA. This result suggests that the 40-channel SQUID-MEG system achieves source localization accuracy comparable to that of the 90-channel OPM-MEG system under the experimental and analytical conditions demonstrated in this study.

**Figure 13:**
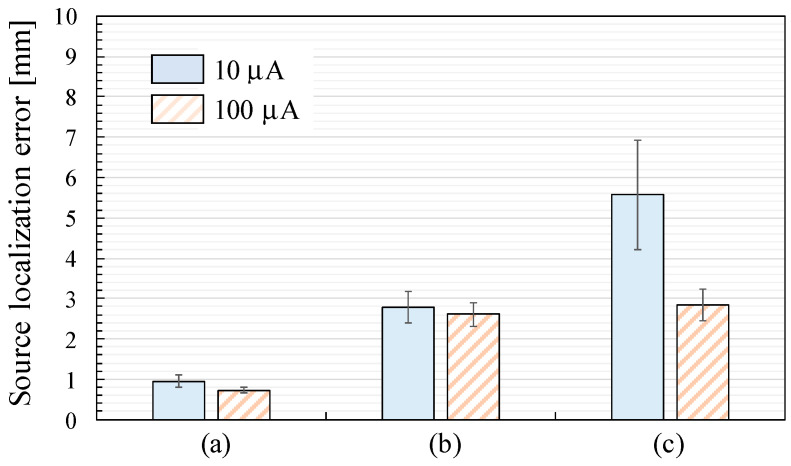
Source localization error defined derived using the data of 35 ECDs excluding the data from 14 ECDs located below the rim of the sensor array: (**a**) SQUID-MEG system; (**b**) OPM-MEG system (closest position); (**c**) OPM-MEG system (lift-off position).

Third, the source localization accuracy is also influenced by the spatial coverage provided by the sensor array on the phantom source signal. As presented in Figure 5b,c, some ECDs were positioned below the rim of the sensor array. Therefore, we re-calculated the mean and expanded uncertainty of the source localization error using the estimated results of 35 ECDs, excluding the data from 14 ECDs located below the rim of the sensor array. Figure 13 shows the source localization error derived from the data of 35 ECDs. Comparing the results derived from the data of 49 ECDs shown in Figure 10, the source localization error was slightly decreased when using sensor arrays (b) and (c) with a current amplitude of 10 µA. This result highlights the need for the careful design of the sensor arrangement to ensure the spatial coverage of the signal source, even though the OPM sensor can be easily moved and positioned closer to the scalp compared to the SQUID sensors. However, the OPM-MEG system still exhibited a larger source localization error than the SQUID-MEG system, as shown in Figure 13.

As stated above, we investigated three major factors contributing to source localization error: SNR, the number of sensors used for estimation, and spatial coverage provided by the sensor array on the phantom source signal. Although the spatial coverage of the sensor array on the signal source can improve the source localization accuracy, none of the three factors emerged as the dominant determinant of localization accuracy in the experiments. Several additional factors may restrict the source localization accuracy of MEG systems. One possible reason is a calibration error in the OPM sensor array. Defining sensor arrangements (position and orientation) is essential for precise source estimation. We previously reported that calibrating sensor position, orientation, and sensitivity (converting coefficient from voltage to magnetic flux density) could improve source localization accuracy in biomagnetic measurement systems. This improvement has been demonstrated in a whole-head SQUID-MEG system [29] and a magnetocardiography system using magnetoresistive sensors that operate at room temperature [31]. The SQUID-MEG system sensor array used in this study was calibrated with multiple coils during installation ten years ago. Conversely, the OPM sensor arrangement was determined as the set containing the sensor positions (three-dimensional points, from which the depth of the sensor can be deduced) and the sensor orientation vectors. The values for each component of the sensor arrangement are provided by the constructor of the OPM-MEG system. Recent studies have shown that calibrating OPM sensor positions can improve source localization [8,32]. To improve OPM-MEG system performance, ongoing research focuses on refining sensor placement using our calibration technique.

The phantom-based approach offers a controlled environment where the amplitude, location, orientation, and timing of the source signal can be precisely set and repeated as needed without interference from physiological artifacts such as eye blinks, muscle activity, or cardioballistic noise. As OPM-MEG systems become widespread, researchers may seek to evaluate their performance under different sensor arrangements and/or numbers. This quantitative validation method provides a standardized benchmark for comparing MEG systems across different setups and constructors worldwide.

## 5. Conclusions

We used a dry phantom to compare the source localization accuracy of SQUID- and OPM-MEG systems. Positioning the OPM sensors closer to the signal source resulted in a signal amplitude approximately 3–4 times larger than that detected by the SQUID-MEG system. The displacement between the effectual and estimated ECDs was 1.07 ± 0.17 and 3.48 ± 0.58 mm in the 202-channel SQUID- and 90-channel OPM-MEG systems, respectively.

## Figures and Tables

**Figure A1:**
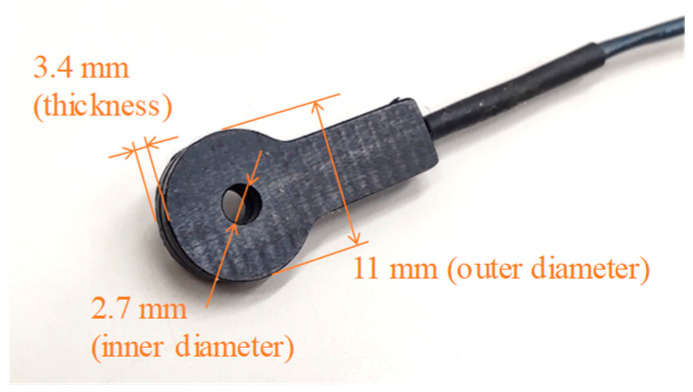
Photograph and dimensions of the marker coil used in this study.

**Figure A2:**
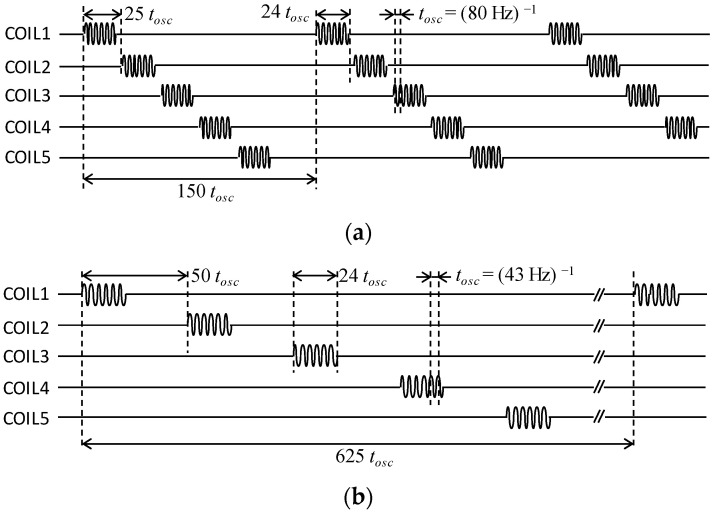
Sequence of current application to the marker coils for the experiments with (**a**) SQUID-MEG system and (**b**) OPM-MEG system. In the same way as in Figure 6b, *t_osc_* is the reciprocal of the frequency of the oscillator of the current-driver circuit.

## Data Availability

Data are available from the corresponding author upon request via e-mail.

## Abbreviations

The following abbreviations are used in this manuscript:

MEG	Magnetoencephalography
SQUID	superconducting quantum interference device
OPM	optically pumped magnetometer
ECD	equivalent current dipole
SNR	signal–to–noise ratio
MSR	magnetically shielded room

## Appendix A

As described in Section 2.3, five marker coils were used to measure the position of the phantom. Figure A1 shows a photograph of the marker coil along with its dimensions. This coil is typically used with the SQUID-MEG system at NYUAD and is fixed on the surface of the phantom by inserting it into a cylindrical marker pin with a diameter of 2.5 mm. Although OPM-MEG systems include marker coils, the coils are incompatible with the cylindrical marker pins due to differences in diameter. Therefore, we used the SQUID-MEG system marker coils for this study. Figure A2 shows the sequence for applying a sinusoidal current to the marker coils. For the SQUID-MEG system experiment, we used a conventional current-driving circuit with a signal frequency of 80 Hz. Conversely, a custom-built current-driving circuit was developed for the OPM-MEG system experiments. This circuit, designed initially to drive 24 coils with adjustable frequency settings, was configured to match the marker signal frequency of the OPM-MEG system (HEDscan, FieldLine Medical Inc., Boulder, CO, USA), which operates at 43 Hz [24].
